# An analysis of qualitative and mixed methods abstracts from Japanese, UK and US primary care conferences

**DOI:** 10.1186/s12930-018-0048-8

**Published:** 2018-11-22

**Authors:** Makoto Kaneko, Takuya Aoki, Ryuichi Ohta, Machiko Inoue, Rakesh N. Modi

**Affiliations:** 1 Department of Family and Community Medicine, Hamamatsu University School of Medicine, 1-20-1, Handayama, Higashi-ku, Hamamatsu, 431-3192 Japan; 2 Shizuoka Family Medicine Training Program, 1055-1, Akatuchi, Kikugawa, Shizuoka, 437-1507 Japan; 3 Department of Healthcare Epidemiology, School of Public Health in the Graduate School of Medicine, Kyoto University, Yoshida-Konoe-cho, Sakyo-ku, Kyoto, 606-8501 Japan; 4 Faculty of Community Care, Unnan City Hospital, 96-1 Iida, Daito-cho, Unnan, Shimane 699-1221 Japan; 5 Department of Primary Care and Population Health, University College London, Rowland Hill Street, London, NW3 2PF UK; 6 Institute for Global Health, Institute of Child Health, University College London, 30 Guilford Street, 3rd Floor, London, WC1N 1EH UK; 7 Junior International Committee, Royal College of General Practitioners, 30 Euston Square, Kings Cross, London, NW1 2FB UK

**Keywords:** Qualitative research, Primary health care, Japan, United Kingdom, United States

## Abstract

**Background:**

As research in family medicine covers varied topics, multiple methodologies such as qualitative research (QR) and mixed methods research (MMR) are crucial. However, we do not know about the difference in the proportion of QR or MMR between Japan, the UK and the US. This knowledge is needed to shape future research within countries with developing primary care such as Japan and other Asian countries. This study aims to describe the use of QR and MMR in Japanese primary care and compare this to the UK and US; then to make informed recommendations for primary care research.

**Methods:**

A repeated cross-sectional study (2012–2016) based on the abstracts submitted to the annual conferences of the Japanese Primary Care Association in Japan, the Royal College of General Practitioners in the UK, and the North American Primary Care Research Group in the US and other North American countries. The proportions of QR/MMR among all the posters and paper presentations for each of these three conferences were assessed. Also examined were trends and types of qualitative techniques for all three countries and participants/settings for Japan.

**Results:**

There were 1080 abstracts for Japan, 575 for UK and 3614 for US conferences. QR/MMR proportions were 7.5%, 15.1% and 28.1%, respectively. Japan’s proportion was lower than that of UK and US (p < 0.001). The proportion was increasing over time for the UK (p = 0.02). Steps for coding and analyses was most popular for Japan, thematic analysis for the UK and grounded theory for the US. Primary care doctors and hospitals were the commonest contexts for Japan.

**Conclusions:**

QR and MMR were not as popular in primary care in Japan compared to the UK and the US, whereas their use was increasing in the UK. Approaches, participants and settings may differ among these countries. Education and promotion of QR/MMR and multi-disciplinary collaborations need to be recommended in Japan with developing primary care.

## Background

Quantitative research had been predominant in health care research for a long time. Recently, however, the importance of qualitative research (QR) has been recognized as it contributes to deeper understanding and interpretation of the meaning of phenomena in the real world [1]. In addition, mixed methods research (MMR), which combines and integrates quantitative methods with qualitative methods, has become popular in order to capitalize on the advantages of both methods [1].

Although QR and MMR were more often used in nursing disciplines compared to medicine [2], QR and MMR are indispensable for family medicine. The reason is that family medicine is, according to Miller and Crabtree, “a clinical domain where balancing qualitative and quantitative research styles benefits both patients and health care professionals” (A quantitative analysis of qualitative studies in clinical journals for the 2000 publishing year page 2) [2]. Moreover, research in family medicine also encompasses a variety of topics so that multiple methodologies are particularly crucial [3]. Therefore, researchers in family medicine need to acquire proficiency in QR and MMR skills.

In countries with long-established family medicine systems such as the United Kingdom (UK) and the Unites States (US), QR and MMR have become popular. For instance, in the UK, a quarter of submissions to the British Journal of General Practice used qualitative methods with a similar acceptance rate to quantitative studies and these articles were highly cited [4]. In addition, in the US, the Society of Teachers of Family Medicine stressed the importance of qualitative methods in family medicine education [5]. On the other hand, in Japan, family medicine has not been well established, where physicians who received an internal medicine based training program have played a principal role in the primary care setting [6] and the system of certification for family physicians as a new category of specialist has just established since 2017 by an independent third-party organization [7].

Although the editorial board of An Official Journal of the Japan Primary Care Association is planning to develop a guideline for submitting qualitative research [8], in countries with developing family medicine systems such as Japan and other Asian countries, the importance of QR and MMR is not emphasized. This is important to know for these key research-leading countries to enable collaboration with Japanese and other Asian family physicians in order to appropriately mold the future of primary care research.

The question is, therefore, how are qualitative and mixed methods used in primary care research in Japan compared to the UK and US in recent years, and what can be done to improve this?

The aim is to describe the proportion, trend, types and characteristics of QR and MMR in primary care research in Japan compared to in the UK and US between 2012 and 2016, using the numbers of abstracts submitted to major annual conferences, and to make recommendations for QR and MMR development based on these results.

## Methods

We conducted a repeated cross-sectional study on the numbers of abstracts accepted to major annual conferences in primary care in Japan, UK, and US

### Subjects

As major academic conferences in primary care, we selected the Japan Primary Care Association (JPCA) annual conference as a representative of primary care in Japan; the Royal College of General Practitioners (RCGP) annual conference for the UK; and the North American Primary Care Group (NAPCRG) annual meeting for the US. The JPCA is the main primary care association in Japan with approximately 11,000 members [9]. The RCGP is the oldest primary care society in the world with 50,000 members [10]. The NAPCRG is the major primary care society in the US and northern American countries [11].

The study subjects were the accepted abstracts for all posters and oral presentations in the annual conferences of the JPCA, the RCGP and the NAPCRG between 2012 and 2016. In April 2017, we searched these associations’ websites and if insufficient information was available, we contacted the associations for the titles and abstracts. We defined the studies using QR and MMR as all posters and oral presentations that included any of the words below in the title or abstract; for study design, “qualitative/qualitative research/survey”, “mixed-methods” and “qualitative and quantitative”; for analytical method, “grounded theory”, “phenomenology”, “ethnography”, “case study”, “discourse analysis”, “narrative”, “KJ method”, “content analysis”, “action research”, “field work”, “life story”, “thematic analysis”, SCAT”, “immersion-crystallization approach” and “constant comparative approach”.

We also categorized the participants, settings and diseases/conditions of the included studies in the JPCA conferences. The details of eligibility criteria and categorization were shown in Table 1. We shared the work of classification/categorization between authors and when the classification of the study was ambiguous, discussion continued until a unanimous consensus was reached.

**Table 1:** Eligibility criteria and categorization of QR and MMR in the study

Conference	The annual conference of the JPCA The annual conference of the RCGP The annual conference of the NAPCRG
Duration	2012–2016
Category of research	JPCA: “Research” “Hinohara prize (research award)”
	RCGP: “Research”, “Clinical”, “Education”
	NAPCRG: all research except preliminary workshop, workshop, forum
Definition of QR and MMR in the study	We defined as QR and MMR all posters and oral presentations that included the words below in the title or abstract Words from Mackibbon et al. [2] “Qualitative/qualitative research/survey”, “mixed-methods”, “qualitative and quantitative” “Grounded theory”, “phenomenology”, “ethnography”, “case study”, “discourse analysis”, “narrative” Words from Saiki et al. [11] “KJ method”, “content analysis”, “action research”, “field work”, “life story” Words added by authors “Thematic analysis”, SCAT”, “immersion-crystallization approach”, “constant comparative approach”
Categorization of characteristics of QR and MMR in the JPCA	Categories based on Mckibbon et al. [2] Participants Patients/family/nurses/other people/physicians/other health care professionals Settings Hospital/clinic/community/nursing home/emergency department Disease/condition Various/cancer/mental health/pregnancy/cerebrovascular disease/general health/frail elderly/HIV/drugs/death and dying/diabetes/critical care/injury/asthma/pain/smoking/miscellaneous disease

*QR* qualitative research, *MMR* mixed methods research, *JPCA* Japan Primary Care Association, *RCGP* Royal College of General Practitioners, *NAPCRG* North American Primary Care Group

### Statistical analysis

We calculated the total proportion of the selected studies that are QR and MMR in each conference; the denominators were the numbers of all studies and the numerators were the numbers of QR and MMR.

We used Chi squared test for the comparison between the proportion in the JPCA annual conference and the RCGP annual conference and, separately, the NAPCRG annual meeting; the null hypothesis was of no difference in proportions. The significance level selected was 5%. We also used the Cochran–Armitage test to assess the annual change in this proportion for each conference; the null hypothesis was that of no change. The significance level selected was 5% with two-sided tests. We assessed the number and proportions of the different types of qualitative approaches used for each country’s conferences. Finally, we assessed the number of eligible studies in each category of participants and settings for the JPCA conferences. The statistical package used was Stata, version 15.

## Results

We searched 1080 studies for the JPCA Annual Conference, 575 for the RCGP Annual Conference, 3614 for the NAPCRG Annual Meeting respectively. The abstracts of the RCGP annual conference 2012 and 2013 were not available despite enquiry and efforts by the office of the RCGP.

The total proportions of QR and MMR were 7.5 (n = 81, 95% CI 5.9, 9.1), 15.1% (n = 87, 95% CI 12.2, 18.5) and 28.1% (n = 1016, 95% CI 19.0, 21.7) for the conferences of the JPCA, RCGP and NAPCRG, respectively for the study period. The proportion of QR and MMR in the JPCA Annual Conference was significantly lower than that in the RCGP Annual Conference (p < 0.001) and the NAPCRG Annual Meeting (p < 0.001). The proportion of QR and MMR in the RCGP Annual Conference increased yearly (p = 0.02) and those of the JPCA Annual Conference and the NAPCRG Annual Meeting displayed no significant changes. Although the proportion of MMR in the US was increasing, the trend was not statistically significant (p = 0.054).

Table 2 displays these results and the breakdown of studies into QR or MMR. QR was more often conducted in comparison with MMR.

**Table 2:** The proportion of posters and oral presentations that were QR or MMR in each conference and the annual change

Year	2012	2013	2014	2015	2016	Total
JPCA						
All research	247	170	227	208	228	1080
QR	17	11	12	11	17	68
MMR	3	2	5	2	1	13
Proportion that are QR or MMR (%)	8.1	7.6	7.5	6.2	7.9	7.5
95% CI						5.9–9.1
RCGP						
All research			241	170	164	575
QR			26	17	28	71
MMR			1	6	9	16
Proportion that are QR or MMR (%)			11.2	13.5	22.6	15.1
95% CI						12.2–18.1
NAPCRG						
All research	569	653	838	715	839	3614
QR	142	94	183	182	134	735
MMR	41	43	59	66	72	281
Proportion that are QR or MMR (%)	32.2	21.0	28.9	34.7	24.6	28.1
95% CI						26.7–29.6

*QR* qualitative research, *MMR* mixed method research, *JPCA* Japan Primary Care Association, *RCGP* Royal College of General Practitioners, *NAPCRG* North American Primary Care Group, *CI* confidence interval

**Fig. 1:**
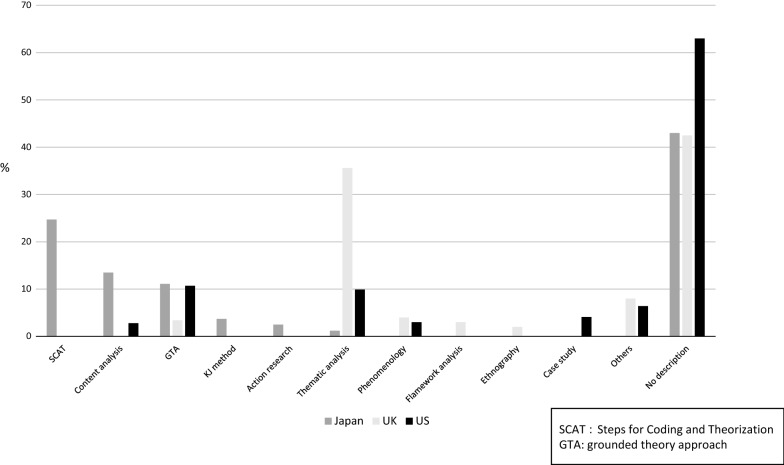
Qualitative approach for QR and MMR studies at each country’s conference

The most frequently used qualitative method in the JPCA Annual Conference was Steps for Coding and Analyses (SCAT), thematic analysis in the RCGP Annual Conference, and grounded theory in the NAPCRG Annual Meeting (Fig. 1).

In QR and MMR in Japan, family physicians were the commonest participants (31 studies). Other common participants were patients (9) and nurses (6). The most frequent study setting was hospital (29), followed by community (24) and clinic (20). In terms of diseases/conditions, almost all of the studies were classified in Various. Other diseases/conditions were mental health (2), death and dying (2) and frail elderly (2).

## Discussion

In comparing the numbers of abstracts submitted to major annual conferences in primary care, the proportion that were QR or MMR in Japan were lower than those in the UK and the US. The trend was not changing in recent years, but it was increasing in the UK. SCAT was the most popular qualitative approach for primary care research in Japan, thematic analysis in the UK and grounded theory in the US.

Our finding of the low proportion of QR and MMR in Japanese primary care may be explained by insufficient development of clinical research in primary care [12], low awareness of the significance of QR and MMR, and lack of educational resources such as mentors. In addition, reporting of QR and MMR studies in the English language may be difficult to perform for Japanese researchers compared to quantitative studies because QR and MMR conducted in Japanese may be harder to translate into English for publication due to a higher reliance on complex language skills. English language QR and MMR may also, therefore, be harder to understand for Japanese researchers, which may discourage learning about and specializing in QR and MMR. Saiki, however, indicated that QR was increasing annually in Japanese health-care journals overall and the number of QR studies had increased 10-fold in the past 10 years [13], which was explained by an increase in Japanese textbooks about QR and MMR [13]. Therefore, although our study revealed no significant change in the proportion of QR and MMR in primary care, these research methods may become popular in the near future. Based on the results of our study and the current literature, it is necessary to examine further the barriers and facilitators for QR and MMR in Japan and other Asian countries with developing primary care. In the UK, large organizations have mentioned that QR/MMR are essential to primary care research, e.g. the Academy of Medical Sciences in their report on research in general practice [14]. Also, in line with this, almost all primary care departments have qualitative research groups or forums to foster these skills amongst new researchers, which expands the research base, e.g. Oxford and Cambridge [15, 16]. These factors may affect the increase of the proportion of QR/MMR in the results.

We also do not know if the proportions of QR and MMR in the UK and US are sufficient or appropriate for the needs of their primary care systems. The proportion of QR and MMR is not the only indicator of the influence of qualitative and mixed methods; we also need to consider the quality of research and relevance to clinical practice.

In addition, the most frequently used qualitative method in the JPCA was SCAT. SCAT is a qualitative analytical method developed by Ohtani, Japan [17]. The method consists of 4-step coding and description of storyline, suitable for the analysis of small-size qualitative data and easy to use for novices [17]. The SCAT website has described “how to use SCAT”, “Frequently Asked Questions” and “Tips and Pitfalls” for free in Japanese [17]. Also, the author of SCAT has provided family physicians with workshops overall Japan [17]. Moreover, some research course for family physicians have included a lecture series on SCAT [18]. These rich resources may explain why it is more widely understood and used in Japan. Provision of such resources in other methods may be helpful to distribute QR/MMR among Japanese family physicians. This study also found that, in Japan, family physicians were the commonest participants in QR and MMR, and hospitals were the most frequent study settings. The results may be explained by the fact that almost of all members of the JPCA were family physicians [9] and 43.7% of the JPCA certified family physicians work not only at clinics but also at hospitals [19].

In contrast, according to an analysis of QR in 170 English language journals, patients and family were the first and second most common types of participants, and the community was the commonest setting [2]. They also found that half of the qualitative studies were conducted by nurses [2]. This situation is similar in Japan, where most QR is published in nursing journals [20]. In order to enhance the use of QR and MMR for patient-centered care in Japanese and other Asian countries’ primary care, multi-disciplinary collaborations would be useful.

To deal with the wide-range research questions in primary care, both qualitative and quantitative methods play important roles [3]. In Japan, Aomatsu pointed out that the qualitative method is indispensable to deeply understand the problems of patients with respect to not only the biological phenomena but also the psycho-social and contextual phenomena in primary care settings [8]. QR and MMR are also useful to describe and understand complex issues without an over-simplification and can identify interactive relationships between problems [8].

### Strengths and implications

This study covers a largely unexamined issue that is important for the global development of primary care via research. The large sample of studies over time and the use of abstracts of conference posters and presentations had possibly increased the representativeness of our findings for the general state of primary care research in these countries. The examination of the characteristics of qualitative research illustrated the need to further describe the problem, and by this, seek possible solutions for the underuse of qualitative research. The following lessons could be learned for Japan and other Asian countries from the pattern of UK and US primary care research: education and promotion of QR and MMR are highly recommended in Japanese and other Asian countries’ primary care, which could be achieved by promoting domestically developed analytical methods, supporting translations of their studies reported in non-English languages into English for publication, and multi-disciplinary collaborations. QR and MMR may also be made more relevant by focusing on patients and the community instead of secondary care settings.

### Limitations

We only included presented posters and oral presentations, therefore the data from workshops, reports and others were not used, which may have caused selection bias. We may therefore have underestimated the use of QR and MMR in Japan. Moreover, we did not differentiate poster presentations and oral presentations in the process of data collection. Thus, this study could not compare the proportion of oral presentations of QR/MMR with other study designs. We also assumed conferences from the JPCA, the RCGP and the NAPCRG were representative of each country’s primary care research. The selection of the conferences was based on our knowledge and information from their websites, but selection bias may have been introduced by not examining other conferences. If other conferences about QR/MMR and primary care included more studies with QR/MMR in primary care, we might have underestimated the proportion of QR and MMR conducted in these countries. Moreover, preference and knowledge about QR/MMR among reviewers in these conferences may have influenced the results. Also, we reviewed only abstracts of the studies, and sometimes the information about the analytic approach was not included. Thus, our results did not necessarily describe the precise picture of the analytic approach due to its lack of description in conference abstracts. The results therefore need careful interpretation. Moreover, as the nationalities of researchers were not known from the title/abstracts, this study assumed that authors were from the countries in which the conferences were based. As such, there is a possibility that the research conducted by authors from other countries could have been included in the conference abstracts, therefore introducing misclassification. However, we excluded “international session” from our study. Thus, the proportion of studies by other countries’ researchers may not change our results. Lastly, we shared the work of classification/categorization, and it could have led to the variances of classification.

## Conclusions

QR and MMR were not highly prevalent in primary care within Japan compared to the UK and the US, whereas their use was increasing in the UK. Approaches, participants and settings may differ among these countries. Education and promotion of QR/MMR and multi-disciplinary collaborations should be recommended in Japan.

## Abbreviations

QR: qualitative research
MMR: mixed methods research
JPCA: Japan Primary Care Association
RCGP: Royal College of General Practitioners
NAPCRG: North American Primary Care Research Group
SCAT: steps for coding and analyses

## References

[1] Liamputtong P (2016). Research methods in health: foundations for evidenced based practice.

[2] McKibbon KA, Gadd CS (2004). A quantitative analysis of qualitative studies in clinical journals for the 2000 publishing year. BMC Med Inform Decis Mak.

[3] Greenhalgh T (2008). Research methods for primary health care.

[4] Sidhu K, Jones R, Stevenson F (2017). Debate & analysis: publishing qualitative research in medical journals. Br J Gen Pract.

[5] Stumbar S, Brown D. Qualitative Research in Family Medicine Education. The society of teachers of family medicine. 2016. http://www.stfm.org/NewsJournals/EducationColumns/October2016EducationColumn. Accessed 23 Apr 2018.

[6] Kusaba T, Yokobayashi K (2013). Comprehensiveness and education about common disease in outpatient care of primary care clinics. Recommendations: common disease in Japan generalist teachers consortium.

[7] Overview of training program for general practitioners. The Japanese Medical Speciality Board. 2017. http://www.japan-senmon-i.jp/comprehensive/index.html. Accessed 8 Aug 2018.

[8] Aomatsu M (2017). Qualitative research in primary care and effort of editors. Off J Jpn Prim Care Assoc.

[9] The Japan Primary Care Association: About us. The Japan Primary Care Association. 2017. http://www.primary-care.or.jp/. Accessed 23 Apr 2018.

[10] Royal College of General Practitioners: Home. Royal College of General Practitioners. 2017. http://www.rcgp.org.uk/. Accessed 23 Apr 2018.

[11] North American Primary Care Research Group: Home. North American Primary Care Research Group. 2017. http://www.napcrg.org/. Accessed 23 Apr 2018.

[12] Aoki T, Fukuhara S (2017). Japanese representation in high-impact international primary care journals. Off J Jpn Prim Care Assoc.

[13] Saiki-Craighill S (2014). Overview of grounded theory approach. Keio SFC J.

[14] Research in general practice: bringing innovation into patient care Workshop report. the Academy of Medical Sciences. 2009. https://acmedsci.ac.uk/file-download/35182-12569153801.pdf. Accessed 31 Aug 2018.

[15] Health Experiences Research Group. University of Oxford. 2018. https://www.phc.ox.ac.uk/research/health-experiences. Accessed 31 Aug 2018.

[16] Qualitative Research Forum. University of Cambridge. 2018. https://www.phpc.cam.ac.uk/pcu/research/qrf/. Accessed 31 Aug 2018.

[17] Ohtani H. SCAT: steps for coding and theorization qualitative data analysis method. 2017. http://www.educa.nagoya-u.ac.jp/~otani/scat/. Accessed 23 Apr 2018.

[18] Jikei Clinical Research Program for Primary Care. Division of Clinical Epidemiology, Jikei Unicetsity School of Medicine. 2018. http://www.jikei.ac.jp/ekigaku/medical/. Accessed 31 Aug 2018.

[19] Toi T, Murata A, Ota H, Ohashi H, Kusaba T (2016). Research on actual condition of family physician in Japan. Off J Jpn Prim Care Assoc.

[20] Kitajima Y, Nishihira T, Nishitani M, Tao M, Miyashiba T, Sakashita R (2012). The current state of and issues in nursing research carried out by clinical nurses with reference to papers in academic journals. RINCPC Bull.

